# Differences in Accumulation of Rare Earth Elements by Plants Cultivated in Soil and Substrates from Industrial Waste Materials

**DOI:** 10.3390/plants14040589

**Published:** 2025-02-14

**Authors:** Dominika Gmur, Grzegorz Siebielec, Monika Pecio

**Affiliations:** Department of Soil Science and Environmental Analyses, Institute of Soil Science and Plant Cultivation State Research Institute, Czartoryskich 8, 21-100 Pulawy, Poland; gs@iung.pulawy.pl (G.S.); mpecio@iung.pulawy.pl (M.P.)

**Keywords:** rare earth elements, phytoextraction, contaminants, plant-based technology, soil remediation

## Abstract

The aim of this experiment was to investigate the differences in the uptake and accumulation of rare earth elements (REEs) between selected plant species and the substrates used (soil with increased REE content, ash, and smelter waste). Eight plant species were included in the study: common yarrow (*Achillea millefolium)*, false mayweed (*Triplerosperum maritimum*), tall fescue (*Festuca arundinacea*), marigold (*Tagetes* sp.), maize (*Zea mays*), white mustard (*Sinapis alba*), red clover (*Trifolium pratense* L.), and autumn fern (*Dryopteris erythrosora*). The study focused on the following REE representatives: lanthanum (La), cerium (Ce), europium (Eu), and gadolinium (Gd). Plant samples, divided into roots and shoots, were analyzed by ICP-MS. The obtained REE concentrations in plant tissues ranged from 9 to 697 µg kg^−1^ (La), 10 to 1518 µg kg^−1^ (Ce), 9 to 69 µg kg^−1^ (Eu), and 9 to 189 µg kg^−1^ (Gd). To determine the ability of plants to phytoextract REE, two factors were calculated: the translocation factor (TF) and the bioconcentration factor (BCF). The highest TF value was obtained for *D. erythrosora* growing on a substrate consisting of soil with increased REE content (Gd, TF = 4.03). Additionally, TF > 1 was obtained for all REEs in *T. pratense* L. In the experiment, the BCF was lower than 1 for all the plants tested. The study provided insight into the varying ability of plants to accumulate REEs, depending on both the plant species and the chemical properties of the substrate.

## 1. Introduction

The inappropriate or uncontrolled management of waste connected with activities such as ore mining and smelting, the chemical industry, the transfer of sewage sludge to agricultural soils, and other waste-generating activities are the processes leading to soil pollution [1]. Mining and mineral processing are responsible for the creation of large amounts of waste materials. This is because only a small percentage of the ore is valuable, so 99% of it is in the form of tailings. Additionally, it is estimated that there are over a million abandoned mines in the world, left without remedial measures. As a result, such types of metal deposits can be used today as a source of secondary resources. Especially for critical metals, demand has been increasing over the years [1,2].

Rare earth elements (REEs) are a group of metals that occur naturally in the environment. They include 15 lanthanides: lanthanum (La), cerium (Ce), praseodymium (Pr), neodymium (Nd), promethium (Pr), samarium (Sm), europium (Eu), gadolinium (Gd), terbium (Tb), dysprosium (Dy), holmium (Ho), erbium (Er), thulium Tm), ytterbium (Yb), lutetium (Lu), yttrium (Y) and scandium (Sc). REEs can be extracted from monazite, bastnaesite, xenotime, and ion-adsorption clays [3].

REEs are commonly found in the Earth’s crust [4]. REE content in soil depends on the minerals in which they are found, soil texture, organic material content, pedogenic processes, and anthropogenic activity. The average REE concentration in the Earth’s crust is about 0.015%, which corresponds to approx. 189 mg kg^−1^; the closer to the Earth’s surface, the lower the content [5].

Due to the properties of REEs, they play an increasingly important role in industry, especially in innovative technologies linked with the trend of reducing the use of fossil fuels. They are used, among others, for the production of wind turbines, hybrid cars, or batteries. REEs are also increasingly used in agriculture as fertilizers [6]. REEs are used in agriculture as growth regulators. They are used in the form of foliar application, fertilizer additives and seed dressings. It has been proven that the use of REEs in crops increased their productivity by 5–15% [7]. But, using REEs in high concentrations has a retarding effect on the growth of plants [8]. In the studies of [9], it was proved that adding fertilizer containing La and Ce to the soil increased the uptake of phosphorus by maize. In the same studies, it was also proved that adding fertilizer in a dose of less than 10 kg/ha containing REEs increased the yield of the tested plant [7]. In turn, after the foliar addition of fertilizer containing REEs to *Triticum aestivum*, an increase in the level of REE accumulation in roots and leaves was observed, whereas no significant presence of these metals was found in the grains [10].

Approximately 95% of all mineral resources containing REEs in the world are contained in three minerals: monazite, xenotime, and bastnasite. The REE content in soils depends on, among other things, the parent material of the soil, climate, weathering intensity or mobility and sequestration of REEs in secondary minerals, and soil organic matter [11]. The effect of the rapid growth in the exploration of mineral resources containing REEs and the increasingly widespread use in modern industry and everyday life is the increase of these metals in ecosystems. The main areas with increased REE content include zones located near polluted sites (large cities or mining and industrial areas), regions where agriculture is intensive, and places where the parent material shows a high REE content [12]. Plants growing in urban or agricultural soils bioaccumulate REE. As a result, excessive content of these metals in the substrate leads to serious consequences for the surrounding environment, including groundwater and agricultural products. The presence of REEs in soil and water can lead to the entry of these elements into the bodies of humans and animals, for example, through consumed food [13]. It has been shown that rare earth mining has affected residents living near mining areas. When analyzing urine for REE content, individuals in these areas showed higher levels of elements compared to those living outside the mining zones [14].

REEs have been classified by the European Union as a group of elements belonging to critical raw materials due to supply risk (CRM). The substitution index (range of 0–1), which is the parameter defining the difficulty of metal substitution, is for REEs assigned as higher than 0.9 [15]. The growing demand for REEs, especially in industry, has led to the release of these metals into the environment. The increasing content of these metals in the environment can have adverse effects on the ecosystem and threaten human health. As a result, it has become crucial to find ways to remediate areas contaminated with REEs and recover them from secondary resources around the world [16].

Traditional methods of remediation of heavy metal contamination are based on physical, chemical, and thermal methods. Physical methods include soil washing, thermal desorption, and replacement or partial replacement of contaminated soil with another soil to reduce the concentration of contaminants. Chemical methods include vitrification technology, chemical leaching, chemical fixation, and the electrokinetic method. These methods are time-consuming and economically unprofitable; some are very complicated and have many limitations [17].

Gentle phytoremediation methods can be an alternative to traditional remediation methods. Phytoremediation can be used to treat various pollutants: heavy metals, xenobiotics, pesticides, organic compounds, and toxic aromatic compounds [18]. For this purpose, microorganisms, plants, or microbiological or plant enzymes are used [19]. Phytoremediation relies on natural processes and plants to remove, degrade, or immobilize pollutants [18]. The effectiveness of phytoremediation depends on many biological processes such as plant–microbe interaction, plant ability to take up contaminants from the substrate, translocation and tolerance mechanisms, and plant chelating ability. [20]. The key to remediation is to reduce the solubility of environmental contaminants by changing pH, redox processes, and adsorption of contaminants from contaminated soils. In summary, the mechanisms of phytoremediation in plant organisms include phytoextraction, phytodegradation, rhizofiltration, phytostabilization, and phytovolatilization. [18].

Plants involved in phytoextraction take up metals dissolved in the soil solution. Metals are absorbed by the roots and then transported to other parts of the plant, such as the stems or leaves [21]. The solubility of metals depends on factors such as soil pH, the concentration of other elements in the soil, and the content of organic matter. Soil pH significantly influences the availability of nutrients and the toxicity of metals to plants. For the phytoextraction process, the optimal soil pH is in the lower range of 6–7. Additionally, the uptake of metals by roots is also influenced by plant species and environmental conditions [2,17,21].

The translocation factor (TF) and bioconcentration factor (BCF) are used to describe the bioaccumulation properties of plants. The TF index describes the ratio of metal concentration in plant shoots to metal concentration in roots. Plants with higher TF values have a greater ability to transfer metals from roots to shoots. In turn, the BCF factor is the ratio of the concentration of an element in the plant to the concentration of the element in the soil. In the case of accumulators of a given group of metals, it is recommended that both TF and BAF be higher than 1, then, such plants are called hyperaccumulators. [6]. If the TF and BCF values for a plant are higher than 1, it is suggested that the plant can be used as a bioaccumulator, whereas if the BCF > 1 and TF < 1 are obtained, the plant can be used as a phytostabilizer. In contrast, the plant can be used as a phytoextractor when the BCF < 1 and TF > 1 [21].

The selection of optimal species for the soil remediation process or additives aimed at improving soil conditions is key to effective phytoremediation [22]. The selection of plant species for phytoremediation processes should take into account the ability of the plant to tolerate or biodegrade high concentrations of pollutants, accumulate elements of interest, grow quickly, produce large amounts of biomass, and be resistant to diseases, pests, and difficult environmental conditions [23].

For example, due to their rapid growth, *Typha latifolia*, *Salix* spp., *Dryopteris dichotoma*, and some fern species are among the most important plant species that can be used in REE phytoextraction. In the case of REEs, hyperaccumulators should accumulate more than 1000 µg g^−1^ of metals in their leaves or have a TF greater than 1. [24].

Based on a literature review, in general, there is missing information on comparing the ability of various physiologically different plant species to uptake and accumulate REEs. There might also be an interaction between the plant species potential and the type of soil or substrate used. Therefore, the aim of the study was to investigate the differences in REE uptake and accumulation between selected plant species and the substrates used (soil with increased REE content, ash, and smelter waste). The study focused on the following REE representatives: lanthanum (La), cerium (Ce), europium (Eu), and gadolinium (Gd). The study basically was not aimed at optimizing the REE accumulation levels in plant tissues but rather to compare a wide range of plant species and substrate compositions to produce indications for further research that would be focused on enhancing the effectiveness of phytoextraction.

## 2. Results

### 2.1. Plant Growth

**Figure 1:**
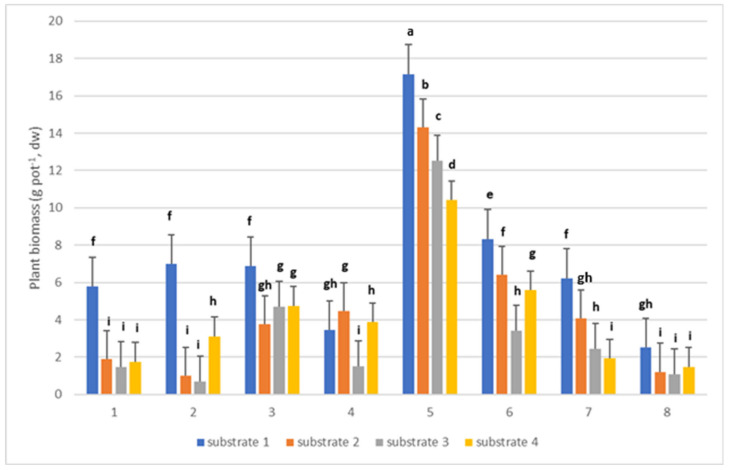
The total biomass production (g pot^−1^, dw, mean± SD, *n* = 3) of 1—*Achillea millefolium*, 2—*Trifolium pratense* L., 3—*Festuca arundinacea*, 4—*Sinapis alba*, 5—*Zea mays*, 6—*Tagetes* sp., 7—*Tripleurospermum maritimum*, 8—*Dryopteris erythrosora* across four substrates: substrate 1 (95% soil, 5% compost), substrate 2 (30% paper industry ash, 20% compost, 50% peat), substrate 3 (30% power plant ash, 20% compost, 50% peat), substrate 4 (40% smelter waste, 20% compost, 40% peat). Values marked with different letters (a, b, c, etc.) for each element in relation to plant species and substrate variants are significantly different at *p* < 0.05 according to Tukey’s HSD test (ANOVA).

The average biomass for each of the eight plant species across the substrates used is presented in Figure 1. Significant differences were observed in plant growth, which strongly depended on the substrate used. For all plants except *S. alba*, the highest biomass was obtained for substrate 1 (soil). For *A. milleflium* it was 5.78 g pot^−1^; for *T. pratense,* L. 6.98 g pot^−1^; for *F. arundinacea,* 6.89 g pot^−1^; for *Z. mays,* 17.15 g pot^−1^; for *T. maritimum,* 6.23 g pot^−1^; for *Tagetes* sp., 8.34 g pot^−1^; and *D. erythrosora,* 2.51 g pot^−1^. For *S. alba*, the highest biomass was observed for substrate 2 and it was 4.47 g pot^−1^. The lowest plant biomass was recorded for substrate 3, where the biomass ranged from 0.69 g pot^−1^ (*D. erythrosora*) to 12.51 g pot^−1^ (*Z. mays*).

### 2.2. Tolerance Index

Table 1 contains data on the calculated tolerance index (TI). The index was calculated as the ratio of the biomass increase in contaminated substrates (i.e., substrates 2, 3, and 4) to the biomass increase in the uncontaminated substrate (substrate 1). The highest TI values were obtained for *S. alba* in substrate 2 (TI = 1.30) and substrate 4 (TI = 1.12). TI values for the remaining plants were below 1. *T. pratense* L. appears to be the least tolerant to contaminants in the substrate, with the lowest TI values of 0.15 (substrate 2) and 0.10 (substrate 3). Additionally, TI values were higher in substrates 2 and 4 compared to substrate 3, with ranges of 0.15–1.30 and 0.30–1.12, respectively.

### 2.3. Change of Substrate pH

Table 2 shows the data for pH measured in H_2_O for the four substrates after plant harvest. The first row presents the initial pH before the establishment of the experiment with plants; the remaining rows contain the results for the individual plant species and substrates.

In substrate 1, the pH values ranged from 5.15 to 5.88; for each plant, the pH was lower compared to the initial state. The pH for substrate 2 ranged from 7.31 to 7.58, for substrate 3 it was 7.55–7.89, and for substrate 4, 7.15–7.26. In the case of the last substrate, the pH was higher than the initial value for each plant. In general, soil pH (substrate 1) decreased during the experiment and plant growth, while the pH of substrates based on industrial wastes was more stable.

Elements are more available to plants when the soil has a lower pH, which affects the bioavailability and mobility of elements in the soil as well as the growth and development of plants. The study confirmed this assumption. In substrate 1, with a lower pH compared to the other substrates, higher yields of the tested plants were obtained. Additionally, plants took up greater amounts of La, Ce, Eu, and Gd compared to other substrates.

### 2.4. Accumulation of REEs in Plant Tissues

**Figure 2:**
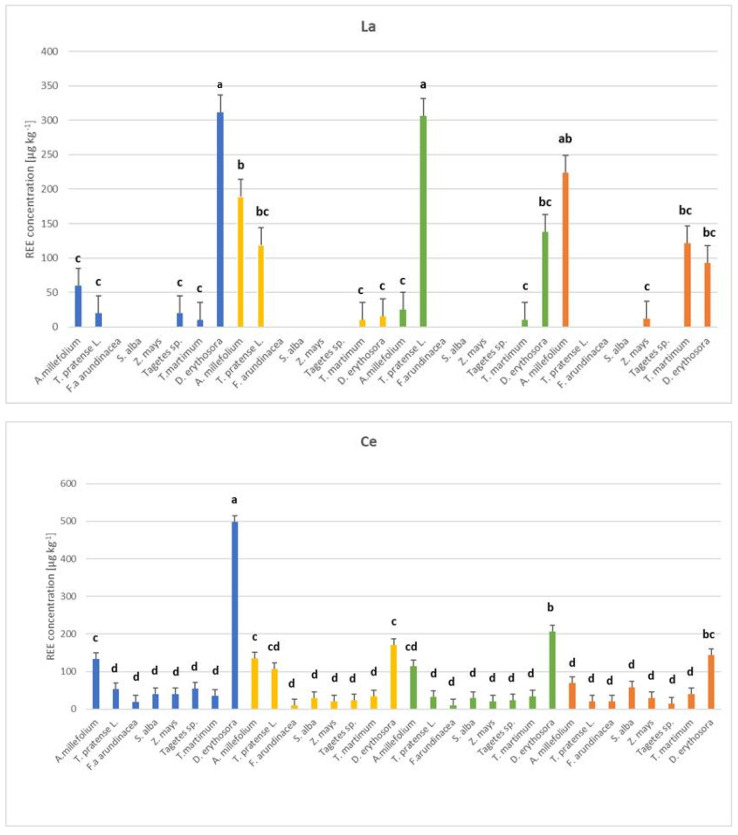

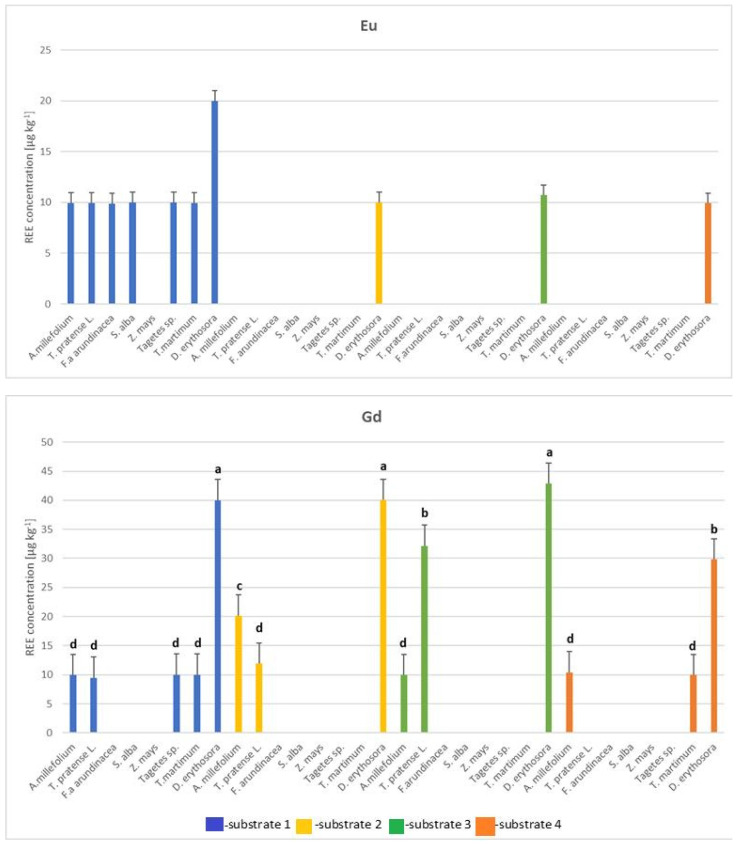
Concentrations of La, Ce, Eu, and Gd in shoots of *Achillea millefolium*, *Trifolium pratense* L., *Festuca arundinacea*, *Sinapis alba*, *Zea mays*, *Tagetes* sp., *Tripleurospermum maritimum*, *Dryopteris erythrosora* across four substrates (µg kg^−1^, mean± SD, *n* = 3). The substrates that were used are as follows: substrate 1 (95% soil, 5% compost), substrate 2 (30% paper industry ash, 20% compost, 50% peat), substrate 3 (30% power plant ash, 20% compost, 50% peat), and substrate 4 (40% smelter waste, 20% compost, 40% peat). Values marked with different letters (a, b, c, etc.) for each element in relation to plant species and substrate variants are significantly different at *p* < 0.05 according to Tukey’s HSD test (ANOVA). No letters indicate no statistical significance at the level of *p* > 0.05. Blank fields indicate results below the detection limit.

The total concentrations of La, Ce, Eu, and Gd in the aboveground parts and roots are presented in Table 2. The results indicate that REEs were accumulated in greater amounts in the roots of plants. The range of La in the aboveground parts was within the range of 9 to 311 µg kg^−1^ for *T. martimum* (substrate 1) and *D. erythrosora* (substrate 1), respectively, while in the root for this element, it was from 9 to 697 µg kg^−1^ for *T. pratense* L. in substrate 1 and substrate 2. The lowest Ce concentration was 10 µg kg^−1^ for *F. arundinacea* (substrate 2), and the highest was 497 µg kg^−1^ for *D. erythrosora* (substrate 1). In the roots, the range was 29–1518 µg kg^−1^ for *T. pratense* L. in substrates 1 and 2, respectively (Figure 2).

**Figure 3:**
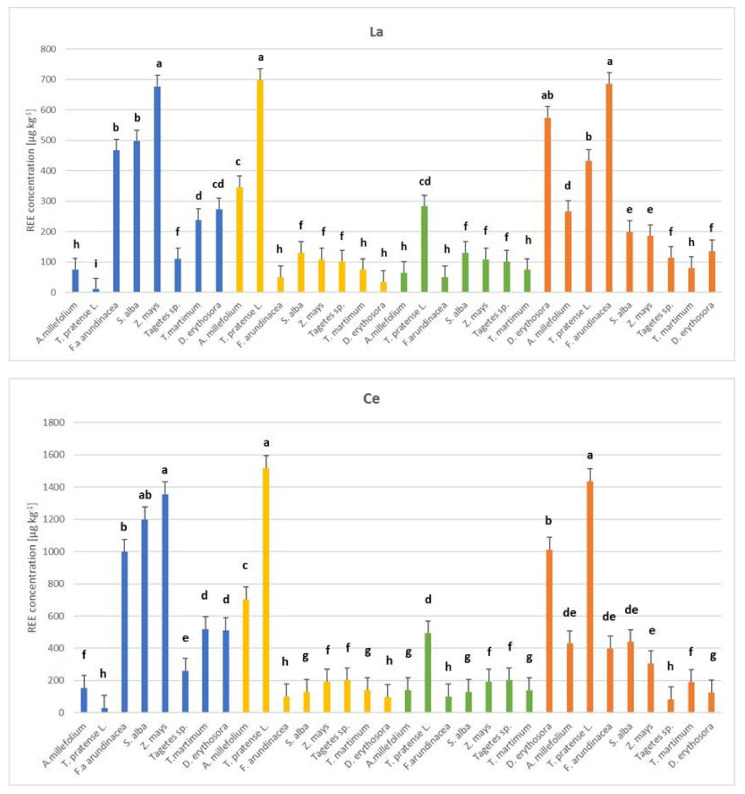

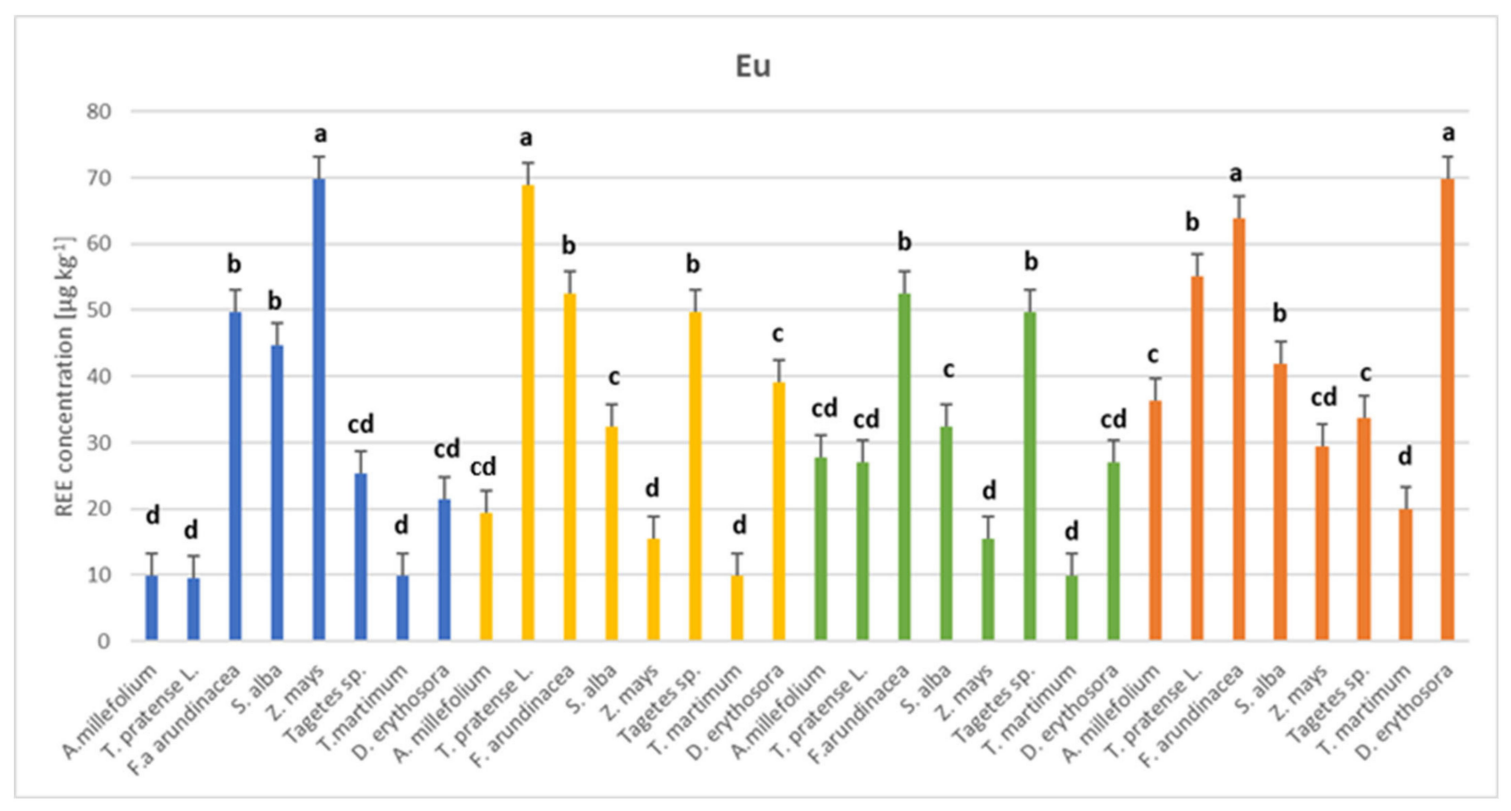

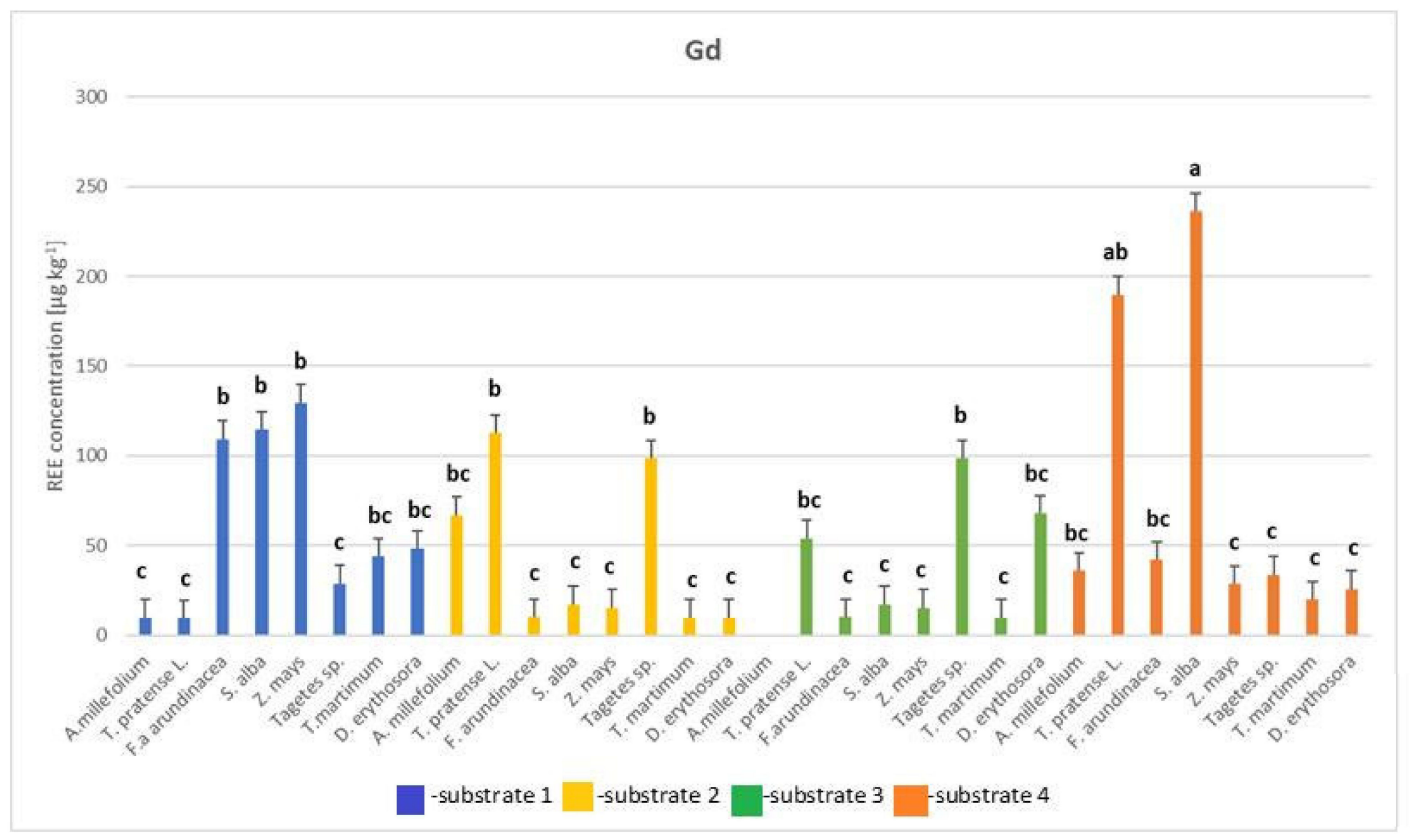
Concentrations of La, Ce, Eu, and Gd in roots of *Achillea millefolium*, *Trifolium pratense* L., *Festuca arundinacea*, *Sinapis alba*, *Zea mays*, *Tagetes* sp., *Tripleurospermum maritimum*, and *Dryopteris erythrosora* across four substrates (µg kg^−1^, mean± SD, *n* = 3). The substrates that were used are as follows: substrate 1 (95% soil, 5% compost), substrate 2 (30% paper industry ash, 20% compost, 50% peat), substrate 3 (30% power plant ash, 20% compost, 50% peat), and substrate 4 (40% smelter waste, 20% compost, 40% peat). Values marked with different letters (a, b, c, etc.) for each element in relation to plant species and substrate variants are significantly different at *p* < 0.05 according to Tukey’s HSD test (ANOVA). Blank fields indicate results below the detection limit.

The Eu range for the aboveground parts was 9 to 20 µg kg^−1^, where the lowest concentrations were detected for *A. millefolium* (substrate 1), *T. pratense* L. (substrate 1), *F. arundinacea* (substrate 1), *T. martimum* (substrate 1), and *D. erythrosora* (substrate 4), and the highest for *D. erythrosora* (substrate 1). For the root, the Eu content ranged from 9 to 69 µg kg^−1^ for *T. pratense* L. (substrate 1) and *D. erythrosora* (substrate 4). Gd contents for aerial parts ranged from 9 to 42 µg kg^−1^ for *T. pratense* L. (substrate 1) and *D. erythosora* (substrate 3), respectively. In turn, Gd content in the root ranged from 9 to 189 µg kg^−1^ for *T. pratense* L. in substrate 1 and substrate 4, respectively (Figure 3).

Two-way analysis of variance revealed a significant (*p* < 0.05) effect of plant species and substrate types on all analyzed elements in individual plant parts. In the case of substrate 1, the lowest REE content was maintained for *T. pratense* L. in the aboveground parts and root (Gd—9 µg kg^−1^ dw). The highest content was obtained for *D. erythrosora* (root, Ce—512 µg kg^−1^ dw) and *T. martimum* (root, Ce—518 µg kg^−1^ dw). On the other hand, for substrate 2, the lowest content—9 µg kg^−1^ dw was obtained in the root for Eu and Gd for *T. martimum,* and Gd for *D. erythrosora*, while the highest was for *T. pratense* L. (root, Ce—1518 µg kg^−1^). In substrate 3, the lowest REE values were obtained for *A. millefolium* (aboveground parts, Gd—9 µg kg^−1^ dw) and *T. martimum* (root, Eu and Gd—9 µg kg^−1^ dw). The highest content was obtained for *D. erythrosora* (root, Ce—1012 µg kg^−1^ dw). In substrate 4, the REE content ranged from 9 to 1436 µg kg^−1^ dw for *D. erythrosora* (aboveground parts, Gd) and *T. pratense* L. (root, Ce), respectively.

### 2.5. Bioconcentration Factor and Translocation Factor

Table 3 presents the bioaccumulation indexes for plant species across the substrates tested. The BCF was lower than 1 in each case. This means that the plants used in the experiment cannot be classified as REE phytoextractors.

The lowest index for La, Ce, and Gd was characteristic of *A. millefolium* (substrate 3) BCF= 0.001; 0.002 and 0.0007, respectively. The lowest index for Eu was obtained for *S. alba* (substrate 3)—0.0088. On the other hand, the highest BCF values were calculated for *Z. mays* in substrate 1 (La BCF = 0.10; Ce BCF = 0.10; Eu BCF = 0.47) and *S. alba* in substrate 4 (Gd BCF = 0.15).

Table 4 presents the translocation factor of all tested plant species on four substrate variants. The TF > 1 for all tested REEs was obtained only for *T. pratense* L. in substrate 1 (La TF = 2.00, Ce TF = 1.78, Eu TF = 1.05, Gd TF = 1.00). The highest TF for La was obtained for *T. pratense* L. in substrate 1 (TF = 2.00), while for Ce, it was TF = 1.80 for *D. erythrosora* (substrate 2). *T. pratense* L. (substrate 1) was characterized by the highest TF for Eu (TF = 1.05). The highest TF for Gd was obtained for *D. erythrosora* in substrate 2 (TF = 4.03), and it was the highest translocation factor obtained in the experiment.

## 3. Discussion

Plant biomass is one of the factors determining the suitability of a given plant species for phytoremediation. In our experiment, the highest biomass was obtained for corn regardless the substrate used. However, the greatest growth of corn was observed on substrate 1. Apparently soil provided better growth conditions than the other substrates with a substantial share of industrial waste. Corn is considered a model plant due to its high biomass increase or significant tolerance to environmental stresses [25]. Comparing the biomass of all plants used in the experiment in relation to individual substrates, it can be seen that the highest biomass was produced on substrate 1 and the lowest on substrate 3, which was based on the power plant ash. In addition, the plants were characterized by a low tolerance index (TI). The values for most plants were low, which may indicate that the plants are less tolerant to the contaminants present in the contaminated substrates. In the experiment [26], the TI was studied for five plant species growing in soil contaminated with nickel (Ni). The TI range in this experiment varied from 3.04 to 219.78 [26]

Soil pH is one of the key factors determining the solubility and bioavailability of metals in the phytoextraction process. If the pH value drops below 6.0, the solubility and bioavailability of metals increase [27]. According to the literature, pH affects many biogeochemical processes in the soil, which in turn influence plant growth, biomass development, and the availability or mobility of metals. When the substrate has a lower pH, elements tend to be more soluble due to high desorption and low adsorption. However, as pH increases, the tendency for adsorption of elements rises, starting from limited adsorption by soil components to almost complete adsorption within a narrow pH range, known as the pH adsorption edge. Studies have shown that, with increasing soil pH, the solubility of most elements decreases [27,28]. The most efficient substrate in the entire experiment was substrate 1, which had the optimal pH for plant growth in the tested plant species. Adding ash to the substrates limited plant growth and reduced the level of rare earth element uptake by plants (substrate 2 and substrate 3). Factors limiting plant growth, development, and the accumulation of elements from the substrate in such an environment include the unfavorable mechanical composition of ash, high pH, lack of essential nutrients (e.g., N and P), and potentially toxic concentrations of elements such as As, B, Cd, Cu, Hg, Mn, Mo, or Pb [29].

Metalloids such as zinc (Zn), copper (Cu), cadmium (Cd), lead (Pb), and arsenic (As) can adversely affect plant growth and development. Cu and Zn are essential micronutrients for plants, playing key roles in many physiological and metabolic processes. However, at high concentrations, they can cause chlorosis or inhibit growth. Additionally, Cu can interfere with nutrient absorption [30,31,32,33]. On the other hand, Cd, Pb, and As are toxic heavy metals that are not required for plant growth. Among other effects, they inhibit plant growth, cause chlorosis, impair root development, limit biomass production, disturb metabolic processes, and affect cell division, leading to the deformation of plant tissues [34,35,36,37].

High concentrations of these elements may result in weaker plant growth in substrates 2, 3, and 4, as well as a reduced ability of these plants to accumulate rare earth elements (REE) compared to plants growing in substrate 1. Substrate 4, for example, was characterized by higher concentrations of toxic metals than substrate 1 (soil). For instance, the content of As and Pb in substrate 4 was 2724 mg kg^−1^ and 21,783 mg kg^−1^, respectively. In comparison, the content of As and Pb in soil collected from a former industrial area (substrate 1) was 2.02 mg kg^−1^ and 9.37 mg kg^−1^, respectively. Flotation tailings, like ash, were characterized by higher concentrations of toxic metals and a more alkaline pH compared to soil. As a result, plants growing in substrate 4 exhibited a lower degree of REE accumulation.

Low REE concentrations in soil typically stimulate plant growth, while high concentrations have a negative effect on plant development and metal phytoextraction. However, there is limited data on the effects of different REE concentrations, which makes it difficult to clearly compare REE phytoextraction across different plant species [38]. For example, it has been shown that La can induce hormesis in *Oryza sativa* L., *Glycine max* L., and *Vicia faba* L. [8]. The range of REEs measured in plants was 19.6–2267 µg kg^−1^ dry matter. The addition of ash or flotation tailings did not increase the level of REE accumulation in plants. Similarly, the author of [39] conducted studies with the addition of coal fly ash (CFA) to alfalfa (*Medicago sativa* L.) and astragalus (*Astragalus adsurgens* Pall.). It was found that adding CFA to the soil did not always significantly increase REE accumulation in plants compared to those growing in soil alone.

In the study by [40], the accumulation of selected REEs in *Dryopteris erythrosora* grown in soil was examined. It was shown that the fern accumulated La 11.79 mg kg^−1^ d.m., 39.41 mg kg^−1^ d.m., and Gd 1.05 mg kg^−1^ d.m. These values were higher than the REE content measured in the present experiment. In another study, the authors of [41] found that *Phytolacca americana* L., naturally growing in an REE extraction region (REE mine with ion absorption), accumulated up to 1040 mg kg^−1^ of these metals in its leaves. The perennial plant showed a preferential accumulation of heavy REEs in the root absorption process and a preferential accumulation of light REEs in the translocation process from the stem to the leaf [42].

*Dicranopteris linearis* is another example of a natural REE hyperaccumulator. Studies conducted in southern China showed that *D. linearis* could accumulate more than 1000 mg kg^−1^ of REEs from both enriched and unenriched substrates. The study also calculated the BCF, which ranged from 1.11 to 14.8, and the TF, which ranged from 1.31 to 19.6 [43].

REE concentrations in common plants in natural conditions are usually low and usually 10^−3^–10^−1^ μg g^−1^ dw. In mining areas, however, the level of REEs in plant organs is higher due to the higher content of metals in these areas [44]. In our experiment, REE accumulation was higher in roots than in other plant organs. The distribution of metals between the main organs of vascular plants is generally diverse. Usually, the metal content is distributed as follows: roots > shoots > leaves [45]. It has been proven that REE can be accumulated by roots due to similar ionic radii, which they share with calcium. In this case, REEs can replace Ca molecules in several physiological processes, responsible for, among others, plant growth and development [46].

The translocation factor (TF) is a parameter that defines the efficiency of a plant in transferring metals from the roots to the shoots. To assess the plant’s ability to take up elements, the bioconcentration factor (BCF) is calculated based on the ratio between the concentrations of elements in the plant tissues (roots, shoots) and the substrate in which the plant grows. If the ratio of metal content in the root to other plant organs and the ratio of metal content in the plant to the substrate, are greater than 1, the plant can be classified as potentially useful for phytoextraction. In practice, it is possible to collect and remove the aboveground parts of the plant, where metals accumulate, from the site [47].

In the experiment, the highest TF factor was calculated for *Dryopteris erythrosora* in substrate 2 (based on ash from the paper industry) for Gd. This plant showed higher TF values compared to the other plants. *D. erythrosora* is a type of fern. Rare earth elements are typically found in soil as ionic compounds, often bound to minerals or organic matter [48]. Fern roots release organic acids (such as citric acid and malic acid) into the soil, which can help dissolve these REE compounds, making them more available for absorption. Additionally, ferns are more resistant to lanthanide toxicity than other plant groups [49]. Studies [50] have shown that La has a beneficial effect on the growth of *D. erythrosora* and is stored in mesophyll cells. It has also been shown that in the sporophyte, REEs are translocated from the roots to the leaves via xylem sap and are stored in the xylem vascular system [39].

In the case of *T. pratense* L., the TF factor was higher than 1 for all the rare earth elements tested. This suggests that clover is characterized by relatively efficient transport of rare earth elements from the roots to the shoots. Similarly, in studies [51] on the use of *T. pratense* L. for phytoremediation of heavy metals in urban areas, the translocation coefficient (TF) was also higher than 1 for Cr (7.428 and 1.956) and Ni (4.038 and 1.997).

However, in the experiment, the TF > 1 was obtained only for particular plants. In most cases, it was lower than 1. In order to assess the ability of the plant to take up elements, calculations are made of the ratio between the concentrations of elements in the plant body (parts) and the substrate on which it grows [21]. In this experiment, the plants did not show the ability to distribute metals between organs. In each case, REEs were retained at the root level or were not acquired by plants to a larger extent.

Studies [52] showed that REE concentrations in parts of *Tagetes erecta* L. ranged from 0.75 to 20.26 mg kg^−1^. It was also found that Ce was the most abundant REE in the samples, constituting 39 to 45% of the total REE concentration. Additionally, the BCF index for REEs was calculated and found to be lower than 1. This supports the results obtained for *Tagetes* sp. in this experiment, where the BCF was also lower than 1.

In another study [53], an attempt was made to identify native plant species growing in mining areas (lead, silver, and zinc mining) that could potentially be used in phytoremediation techniques. It was shown that the BCF for La and Ce in the roots was higher than the BCF for the shoots in the four species studied: *S. oppositifolia*, *S. tenacissima*, *P. milaceum*, and *A. herba-alba*. The BCF range for La was 0.3–9.9, and for Ce, it was 0.1–4.3. However, a TF > 1 was obtained only for *P. milaceum* for La.

Low values of both the transfer factor (TF) and bioconcentration factor (BCF) in the experiment may have been caused by the properties of the substrates. Adding ash or metallurgical waste to the substrates, despite higher REE content compared to the substrate with soil, did not affect the accumulation of metals by the plants. The low values of the coefficients obtained were likely influenced by several factors, including the alkaline pH of the substrate, the potential toxicity of other metals, competition from nutrients and other metals, and the inactivation of REEs by iron or aluminum compounds. Additionally, rare earth metals are not essential elements for plants. The uptake and accumulation of REEs depend on the plant species and morphology. For instance, some plants secrete low molecular weight organic acids in the soil–root system, which act as chelating agents, increasing the desorption of light REEs and facilitating the uptake of metals from the soil by the plants [54].

## 4. Materials and Methods

### 4.1. Experimental Design

Four substrate variants were used in the experiment. The first variant was represented by soil from Srebrna Góra in Lower Silesian Voivodeship in Poland (50°34′32″ N, 16°39′35″ E). Ashes from the paper industry in Ostrołęka and power plant ash from Upper Silesia were used for producing the second and third variants, respectively. The fourth variant was developed using zinc smelter flotation waste. The substrates were enriched with acidic peat and compost to lower the pH level and enrich with organic matter. The compost was collected from the GWDA company, Piła, Poland. It contained 30.2% organic matter and had a pH of 6.2. It was produced based on a mixture of sewage sludge and selectively collected green municipal, food industry, and agricultural waste. The compost is certified as a soil improver.

The experiment was conducted in 1 kg pots in the greenhouse of the Institute of Soil Science and Plant Cultivation—State Research Institute in Puławy (Poland). The final composition of the substrates in the pots is presented in Table 5.

Eight plant species were used as experimental plants, selected based on available literature: common yarrow (*Achillea millefolium*), false mayweed (*Tripleruosperum maritimum*), tall fescue (*Festuca arundinacea*), marigold (*Tagetes* sp.), maize (*Zea mays*), white mustard (*Sinapis alba*), red clover (*Trifolium pratense* L.), and autumn fern (*Dryopteris erythrosora*). *A. millefolium* and *T. maritimum* are common herbaceous plants occurring in temperate climates, and are resistant to unfavorable climatic conditions. In the studies [55], it was proven that *A. millefolium* is able to accumulate higher zinc (Zn) and cadmium (Cd) content, while the authors of [56] proved the ability of yarrow to phytostabilize in Cu-contaminated soils located in the mining region. On the other hand, naturally growing in the mining area, *T. martimum* showed phytoextraction properties for mercury (Hg). *F. arundinacea* is a perennial grass widely distributed on all continents with a temperate climate, quite commonly used in phytoremediation. It is a plant species widely studied in terms of the accumulation of pollutants from soil [57]. *F. arundinacea* has been shown to accumulate higher concentrations of lead (Pb), zinc (Zn), nickel (Ni), cadmium (Cd), and petroleum hydrocarbons [58,59,60]. *Z. mays*, *S. alba* and *T. pretense* L. are crop plants with high biomass growth. These plants are often used in research as model plants. *T. pratense* L. has demonstrated phytoextraction capabilities for high concentrations of arsenic (As), as well as lead (Pb) and antimony (Sb) [61,62]. In turn, *Z. mays* shows, among others, phytoremediation potential for Cu, Cd, Cr, Ni, and phytostabilization potential for Co [63,64,65,66]. In turn, *D. erythrosora* is a fern from Japan. In the literature, it is presented as a natural REE hyperaccumulator [50,67]. *Tagetes* sp. was selected due to its use in urban areas, and there are also reports in the literature documenting its phytoremediation properties. In studies by the authors of [68,69], *Tagetes* sp. was shown to be an effective phytoextractor for Cd and Zn and an exclusion factor for Pb.

Each substrate and plant variant was represented by three replications in the study. The experiment was conducted in a greenhouse for 3 months starting in May. The plants were watered with distilled water according to current needs.

### 4.2. Analysis of Substrate Components

Samples of each component of the four substrates were dried in an oven, ground, and sieved through a 2 mm sieve. The pH in the water slurry was determined in a ratio of 1:5 (sample-water ratio). Total N and total C were determined on a Vario Macro Cube CN analyzer from Elementar Analysensysteme GmbH (Langenselbold, Germany) (according to the methods: DIN/ISO 13878:1998 [70] for total N and ISO 10694:1995 [71] for total C). Soil and waste samples weighing 0.5 g were digested in aqua regia (HCl-HNO_3_ in a ratio of 3:1) in PFA Teflon vessels in a microwave-accelerated reaction system (MarsXpress; CEM Corp., Matthews, NC, USA). The obtained samples were analyzed using ICP-MS (Agilent 7500ce, Santa Clara, CA, USA). The characteristics of the substrates used in the substrates are given in Table 6.

### 4.3. Plant and Substrate Analysis

After three months, the aboveground parts were cut and the roots were gently separated from the substrate. The plant parts were washed with tap water and then distilled water and dried in a dryer for about 2 days at 50 °C. The dried plants were ground for laboratory analysis.

Plant samples, separately aerial parts and roots (0.5 g), were digested in concentrated HNO_3_ in Teflon PFA vessels in a microwave-accelerated reaction system (MarsXpress; CEM Corp., Matthews, NC, USA). The prepared liquid samples were then analyzed using ICP-MS (Agilent 7500ce, Santa Clara, CA, USA). As a certified reference material, soybean flour (INCT-SBF-4) and mixed Polish herbs (INCT-MPH-2) were used.

The substrates from the experiment were sampled after plant harvest, air-dried in a dryer, ground, and sieved through a 2 mm sieve. Then, the substrates were subjected to pH analysis in a water slurry (1:5 sample–water ratio).

### 4.4. Statistical Analysis

The results were analyzed using factorial analysis of variance (ANOVA). Tukey’s test (HSD) was used to analyze differences between the content of elements.

The tolerance index (TI) is often calculated using the following formula [26]:

(1) $$\mathrm{TI}=(\mathrm{Growth}\;\mathrm{of}\;\mathrm{plantin}\;\mathrm{contaminated}\;\mathrm{soil}/\left(\mathrm{Growth}\;\mathrm{of}\;\mathrm{plant}\;\mathrm{in}\;\mathrm{uncominated}\;\mathrm{soil}\right)$$

Two indices were calculated to assess the accumulation of REEs in plant tissues. The bioconcentration factor (BCF) is a calculated value that indicates the ability of plants to remove metal compounds from the soil or substrate:

(2) $$\mathrm{BCF}=C_{\mathrm{plant}\;\mathrm{organs}}/C_{\mathrm{soil}/\mathrm{substrate}}\;$$

where,

C_plant organs_ is the concentration of metal in collected plant tissues, and

C_soil/substrate_ is the concentration of metal in soil or substrate [21].

In this experiment, the metal content used for BCF calculations was the initial value. Additionally, REE concentrations were calculated proportionally (Table 5) from the metal contents obtained from the substrate component analysis.

The second calculated factor was the translocation factor (TF):

(3) $$\mathrm{TF}=C_{\mathrm{shoot}}/C_{\mathrm{root}}$$

where,

C_shoot_ is the concentration of metals in shoots, and

C_root_ is the concentration of metals in roots.

These factors determine the ability of plants to tolerate and accumulate metals [21].

## 5. Conclusions

This experiment demonstrated that the tested plants generally accumulate REEs in low amounts, with a significant portion retained at the root level. As a result, the recovery of REEs from the aboveground biomass was low. Additionally, based on the calculated BCF and TF coefficients, the plants cannot be classified as REE phytoextractors. However, there were significant differences between individual plant species in terms of biomass production, REE concentrations, and accumulation or translocation patterns. For example, clover showed a greater ability to translocate REEs from the roots to the shoots compared to other plant species. In general, based on data such as biomass growth, metal accumulation, and the calculated coefficients, it can be suggested that the most efficient plants for REE accumulation were *Trifolium pratense* L. and *Dryopteris erythrosora*. The study also showed that, among the different plant species, the most optimal substrate was soil collected from a former industrial site enriched with REEs. From a practical standpoint, further research should focus on optimizing REE uptake by plants by adding chelators to the substrates under controlled greenhouse conditions.

## Figures and Tables

**Table 1:** Tolerance index (TI) for eight plant species: *Achillea millefolium*, *Trifolium pratense* L., *Festuca arundinacea*, *Sinapis* alba, *Zea mays*, *Tagetes* sp., *Tripleurospermum maritimum*, and *Dryopteris erythrosora*. The substrates that were used are as follows: substrate 2 (30% paper industry ash, 20% compost, 50% peat), substrate 3 (30% power plant ash, 20% compost, 50% peat), and substrate 4 (40% smelter waste, 20% compost, 40% peat). The highest TI values are marked in bold.

Species of Plants	Substrate 2	Substrate 3	Substrate 4
*A. millefolium*	0.33	0.25	0.30
*T. pratense* L.	0.15	0.10	0.45
*F. arundinacea*	0.55	0.68	0.69
*S. alba*	**1.30**	0.44	**1.12**
*Z. mays*	0.83	0.73	0.61
*Tagetes* sp.	0.77	0.41	0.67
*T. martimum*	0.65	0.39	0.31
*D. erythosora*	0.48	0.43	0.59

**Table 2:** pH values of the four substrates used in the experiment (pH in H_2_O, mean ± SD, *n* = 3). The substrates used were as follows: substrate 1 (95% soil, 5% compost), substrate 2 (30% paper industry ash, 20% compost, 50% peat), substrate 3 (30% power plant ash, 20% compost, 50% peat), and substrate 4 (40% smelter waste, 20% compost, 40% peat). Values marked with different letters (a, b, c, etc.) for each element in relation to plant species and substrate variants are significantly different at *p* < 0.05 according to Tukey’s HSD test (ANOVA).

Species	Substrate 1	Substrate 2	Substrate 3	Substrate 4
Initial pH	6.60	7.50	7.80	7.10
*Achillea millefolium*	5.15 ± 0.031 k	7.31 ± 0.057 d	7.55 ± 0.059 c	7.18 ± 0.021 f
*Trifolium pratense* L.	5.28 ± 0.065 j	7.55 ± 0.069 c	7.67 ± 0.035 b	7.17 ± 0.032 f
*Festuca arundinacea*	5.33 ± 0.038 ij	7.54 ± 0.026 c	7.69 ± 0.095 b	7.23 ± 0.047 e
*Sinapis alba*	5.88 ± 0.131 g	7.49 ± 0.036	7.75 ± 0.006 a	7.15 ± 0.114 f
*Zea mays*	5.53 ± 0.159 I	7.52 ± 0.026 cd	7.76 ± 0.017 a	7.16 ± 0.021 f
*Tagetes* sp.	5.40 ± 0.057 i	7.55 ± 0.032 c	7.70 ± 0.095 b	7.18 ± 0.025 f
*Tripleurosperm maritimum*	5.67 ± 0.055 h	7.58 ± 0.006 c	7.78 ± 0.020 a	7.15 ± 0.025 f
*Dryopteris erythrosora*	5.83 ± 0.186 g	7.57 ± 0.115 c	7.89 ± 0.044 a	7.26 ± 0.046 e

**Table 3:** Bioconcentriation factor (BCF) for La, Ce, Eu, and Gd for eight species: *Achillea millefolium*, *Trifolium pratense* L., *Festuca arundinacea*, *Sinapis alba*, *Zea mays*, *Tagetes* sp., *Tripleurospermum maritimum*, and *Dryopteris erythrosora* across four substrates. The substrates that were used are as follows: substrate 1 (95% soil, 5% compost), substrate 2 (30% paper industry ash, 20% compost, 50% peat), substrate 3 (30% power plant ash, 20% compost, 50% peat), and substrate 4 (40% smelter waste, 20% compost, 40% peat). The highest BCF values are marked in bold.

Type of Substrate	Species	La	Ce	Eu	Gd
substrate 1	*Achillea millefolium*	0.02	0.02	0.13	0.02
substrate 1	*Trifolium pratense* L.	0.004	0.006	0.13	0.01
substrate 1	*Festuca arundinacea*	0.07	0.07	0.40	0.11
substrate 1	*Sinapsis alba*	0.08	0.09	0.37	0.11
substrate 1	*Zea mays*	**0.11**	**0.10**	**0.47**	**0.13**
substrate 1	*Tagetes* sp.	0.02	0.02	0.23	0.03
substrate 1	*Triplerosperum martimum*	0.04	0.04	0.13	0.05
substrate 1	*Dryopteris erythosora*	0.09	0.07	0.28	0.08
substrate 2	*Achillea millefolium*	0.06	0.04	0.03	0.04
substrate 2	*Trifolium pratense* L.	0.09	0.08	0.12	0.06
substrate 2	*Festuca arundinacea*	0.005	0.005	0.09	0.005
substrate 2	*Sinapsis alba*	0.01	0.008	0.05	0.008
substrate 2	*Zea mays*	0.01	0.01	0.02	0.008
substrate 2	*Tagetes* sp.	0.01	0.01	0.09	0.05
substrate 2	*Triplerosperum martimum*	0.009	0.009	0.01	0.005
substrate 2	*Dryopteris erythosora*	0.005	0.01	0.09	0.02
substrate 3	*Achillea millefolium*	0.001	0.002	0.01	0.0007
substrate 3	*Trifolium pratense* L.	0.008	0.004	0.01	0.006
substrate 3	*Festuca arundinacea*	0.006	0.006	0.03	0.006
substrate 3	*Sinapsis alba*	0.003	0.003	0.008	0.003
substrate 3	*Zea mays*	0.005	0.006	0.06	0.006
substrate 3	*Tagetes* sp.	0.005	0.006	0.03	0.006
substrate 3	*Triplerosperum martimum*	0.008	0.007	0.008	0.007
substrate 3	*Dryopteris erythosora*	0.01	0.01	0.01	0.008
substrate 4	*Achillea millefolium*	0.06	0.03	0.12	0.02
substrate 4	*Trifolium pratense* L.	0.06	0.08	0.18	0.12
substrate 4	*Festuca arundinacea*	0.09	0.02	0.21	0.02
substrate 4	*Sinapsis alba*	0.02	0.02	0.14	0.15
substrate 4	*Zea mays*	0.02	0.02	0.09	0.01
substrate 4	*Tagetes* sp.	0.01	0.005	0.11	0.02
substrate 4	*Triplerosperum martimum*	0.02	0.01	0.06	0.01
substrate 4	*Dryopteris erythosora*	0.03	0.01	0.27	0.03

**Table 4:** Translocation factor for La, Ce, Eu, and Gd for eight species: *Achillea millefolium*, *Trifolium pratense* L., *Festuca arundinacea*, *Sinapis* alba, *Zea mays*, *Tagetes* sp., *Tripleurospermum maritimum*, and *Dryopteris erythrosora* across four substrates. The substrates that were used are as follows: substrate 1 (95% soil, 5% compost), substrate 2 (30% paper industry ash, 20% compost, 50% peat), substrate 3 (30% power plant ash, 20% compost, 50% peat), and substrate 4 (40% smelter waste, 20% compost, 40% peat). TF > 1 is marked in bold. There are b.d. presented in the table in case the concentration for a given plant was below the detection limit.

Type of Substrate	Species	La	Ce	Eu	Gd
substrate 1	*Achillea millefolium*	0.80	0.87	**1.00**	**1.00**
substrate 1	*Trifolium pratense L.*	**2.00**	**1.78**	**1.05**	**1.00**
substrate 1	*Festuca arundinacea*	b.d.	0.01	0.19	b.d.
substrate 1	*Sinapsis alba*	b.d.	0.03	0.22	b.d.
substrate 1	*Zea mays*	b.d.	0.02	b.d.	b.d.
substrate 1	*Tagetes* sp.	0.18	0.21	0.39	0.34
substrate 1	*Triplerosperum martimum*	0.04	0.06	**1.00**	0.22
substrate 1	*Dryopteris erythosora*	**1.17**	0.97	0.93	0.83
substrate 2	*Achillea millefolium*	0.54	0.20	b.d.	0.30
substrate 2	*Trifolium pratense L.*	0.17	0.07	b.d.	0.10
substrate 2	*Festuca arundinacea*	b.d.	0.10	b.d.	b.d.
substrate 2	*Sinapsis alba*	b.d.	0.24	b.d.	b.d.
substrate 2	*Zea mays*	b.d.	0.10	b.d.	b.d.
substrate 2	*Tagetes* sp.	b.d.	0.22	b.d.	b.d.
substrate 2	*Triplerosperum martimum*	0.13	0.24	b.d.	b.d.
substrate 2	*Dryopteris erythosora*	0.47	**1.80**	0.25	**4.03**
substrate 3	*Achillea millefolium*	0.40	0.83	b.d.	b.d.
substrate 3	*Trifolium pratense L.*	**1.13**	0.06	b.d.	0.58
substrate 3	*Festuca arundinacea*	b.d.	0.04	b.d.	b.d.
substrate 3	*Sinapsis alba*	b.d.	0.03	b.d.	b.d.
substrate 3	*Zea mays*	b.d.	0.02	b.d.	b.d.
substrate 3	*Tagetes* sp.	b.d.	0.01	b.d.	b.d.
substrate 3	*Triplerosperum martimum*	0.05	0.01	b.d.	b.d.
substrate 3	*Dryopteris erythosora*	0.24	0.20	0.39	0.63
substrate 4	*Achillea millefolium*	0.85	0.16	b.d.	0.29
substrate 4	*Trifolium pratense L.*	b.d.	0.01	b.d.	b.d.
substrate 4	*Festuca arundinacea*	b.d.	0.05	b.d.	b.d.
substrate 4	*Sinapsis alba*	b.d.	0.12	b.d.	b.d.
substrate 4	*Zea mays*	0.06	0.09	b.d.	b.d.
substrate 4	*Tagetes* sp.	b.d.	0.19	b.d.	b.d.
substrate 4	*Triplerosperum martimum*	**1.56**	0.21	b.d.	0.49
substrate 4	*Dryopteris erythosora*	0.69	**1.16**	0.24	**1.17**

**Table 5:** Composition of the substrates used for the greenhouse experiment (weight percentage).

	Soil	Ash	Smelter Waste	Compost (GWDA)	Peat
Substrate 1	95%	-	-	5%	-
Substrate 2	-	30%	-	20%	50%
Substrate 3	-	30%	-	20%	50%
Substrate 4	-	-	40%	20%	40%

**Table 6:** Content of elements (mg kg^−1^) and other chemical properties of raw substrates: 1—soil, 2—ash from the paper industry, 3—power plant ash, 4—smelter waste. High content of elements is marked in bold.

Element	Soil	Paper Industry Ash	Power Plant Ash	Smelter Waste
Li	2.4	19.2	11.3	3.2
Be	0.1	2.1	0.8	1.5
Al	3674	59,437	15,669	10,064
V	10.5	174.1	35.5	54.7
Cr	7.6	104.7	46.6	41.9
Mn	143	447	631	6228
Fe	4689	54,200	14,031	23,6248
Co	1.6	30.5	15	14.7
Ni	4.3	85.1	53.4	70.3
Cu	3.1	48.4	**498.03**	16.2
Zn	14.3	113.7	892.72	**126,228**
As	2.0	22.8	9.3	**2724.46**
Se	0.1	23.1	1.1	12.2
Sr	0.5	30.9	36.1	3.1
Mo	0.1	2.9	3.7	13.7
Ag	0.1	0.2	4.7	39.8
Cd	0.1	**5.2**	**11.2**	**573.6**
Sn	0.1	2.0	22.6	0.5
Sb	0.0	0.4	16.4	7.2
Ba	19.3	317.9	797.7	50.5
La	6.2	67	8.9	7.0
Ce	13.1	120.4	18.6	16.6
Eu	0.1	2.27	0.5	0.2
Gd	0.9	12.45	1.9	1.5
Tl	0.05	0.64	0.4	19.4
Pb	9.3	40.7	117.4	**21,782.9**
Bi	b.d.	0.7	6.3	0.1
Na	21.1	285	**8770**	62.8
Mg	651	4235	10,300	16,277
K	1209	733	18,455	1721
Ca	987	151,265	130,492	37,220
pH in H_2_O	6.60	7.93	11.26	8.55
EC µS/cm	146	1238	11,080	200
Total nitrogen % N	0.07	0.05	0.02	0.12
Total carbon % C	0.75	3.79	1.78	20.60

b.d.—below detection.

## Data Availability

Dataset available on request from the authors. The raw data supporting the conclusions of this article will be made available by the authors on request.

## Abbreviations

The following abbreviations are used in this manuscript:

BAF	Bioaccumulation factor
CRM	Critical raw material
REE	Rare earth elements
TF	Translocation factor
TI	Tolerance index

## References

[1] Corzo Remigio A., Chaney R.L., Baker A.J.M., Edraki M., Erskine P.D., Echevarria G., van der Ent A. (2020). Phytoextraction of high value elements and contaminants from mining and mineral wastes: Opportunities and limitations. Plant Soil.

[2] Okoroafor P.U., Kunisch N., Epede M.N., Ogunkunle C.U., Heilmeier H., Wiche O. (2022). Phytoextraction of rare earth elements, germanium and other trace elements as affected by fertilization and liming. Environ. Technol. Innov..

[3] Ambaye T.G., Vaccari M., Castro F.D., Prasad S., Rtimi S. (2020). Emerging technologies for the recovery of rare earth elements (REEs) from the end-of-life electronic wastes: A review on progress, challenges, and perspectives. Environ. Sci. Pollut. Res..

[4] Grosjean N., Jean M.L., Berthelot C., Chalot M., Gross E.M., Blaudez D. (2019). Accumulation and fractionation of rare earth elements are conserved traits in the *Phytolacca genus*. Sci. Rep..

[5] Kastori R.R., Maksimović V.I., Putnik-Delić I.M. (2023). Rare earth elements in environment and effect plants- A review. Matica Srp. J. Nat. Sci..

[6] Dinh T., Dobo Z., Kovacs H. (2022). Phytomining of rare earth elements—A review. Chemosphere.

[7] Hu Z.Y., Ritcher H., Sparovek G., Schnug E. (2019). Phycological and biochemical effect of rare earth elements on plants and their agricultar significance: A review. J. Plant Nutr..

[8] Agathokleus E., Kitao M., Calabrese E.J. (2019). Hormetic dose response induced by lanthanum in plants. Environ. Pollut..

[9] Xu X., Wand Z. (2007). Phosphorus uptake and translocation in field grown maize after application of rare earth-containing fertilizer. J. Plant Nutr..

[10] Liang T., Zhang S., Wang L., Kung H.T., Wang Y., Hu A., Ding S. (2005). Environmental biogeochemical behaviors of rare earth elements in soil- plant system. Environ. Geochem. Health.

[11] Sager M., Wiche O. (2024). Rare earth elements (REE): Origins, dispersion, and environmental implications—A comprehensive review. Environments.

[12] Ramos S.J., Dinali G.S., Oliveira C., Martins G.C., Moreira C.G., Siqueira J.O., Guilherme L.R. (2016). Rareearth elements in the soil enviroment. Land Pollut..

[13] Adeel M., Lee J.Y., Zain M., Rizwan M., Nawab A., Ahmad M.A., Shafiq M., Yi H., Jilani G., Javed R. (2019). Cryptic footprints of rare earth elements on natural resources and living organisms. Environ. Int..

[14] Brouzitotis A.A., Giarra A., Libralato G., Pagano G., Guida M., Trifuoggi M. (2022). Toxicity of rare earth elements: An overview on human health impact. Front. Environ. Sci..

[15] Amato A., Becci A., Birloaga I., De Michelis I., Ferella F., Innocenzi V., Ippolito N.M., Pillar C., Gomez J., Vegliò F. (2019). Sustainability analysis of innovative technologies for the rare earth elements recovery. Renew. Sustain. Energy Rev..

[16] Soudek P., Petrova S., Benešová D., Vanek T. (2019). Phytoextraction of toxic metals by sunflower and corn plants. J. Food Agric. Environ..

[17] Nnaji N.D., Onyeaka H., Miri T., Ugwa C. (2019). Bioaccumulation for heavy metal removal: A review. SN Appl. Sci..

[18] Phang L.Y., Mingyuan L., Mohammadi M. (2024). Phytoremediation as a viable ecological and socioeconomic management strategy. Environ. Sci. Pollut. Res..

[19] Gawroński S., Łutczyk G., Szulc W., Rutkowska B. (2022). Urban mining: Phytoextraction of noble and rare earth elements from urban soils. Arch. Environ. Prot..

[20] Sharma S., Tiwari S., Hasan A., Saxena V., Pandery L.M. (2018). Recent advances in conventional and contemporary methods for remediation of heavy metal- contaminated soils. Biotech.

[21] Takarina N.D., Pin D.G. (2017). Bioconcentration Factor (BCF) and Translocation Factor (TF) of heavy metal Mangrove trees of Blankan Fish Farma. Makara J. Sci..

[22] Brunetti G., Farrag K., Soler-Rovira P., Nigro F. (2011). Greenhouse and field studies on Cr, Cu, Pb and Zn pytoextraction by *Brassica napus* from contaminated soils in the Apulia Region, Southern Italy. Geoderma.

[23] Mocek-Płóciniak A., Mencel J., Zakrzewski W., Roszkowski S. (2023). Phytoremediation as an effective remedy for removing trace elements from ecosystems. Plants.

[24] Rabbani M., Taqi Rabbani M., Muthoni F., Sun Y., Vahidi E. (2024). Advancing phytomining: Harnessing plant potential for sustainable rare earth element extraction. Bioresour. Technol..

[25] Chang Q., Diao F., Wang Q., Pan L., Dang Z., Guo W. (2018). Effects of arbuscular mycorrhizal symbiosis on growth, nutrient and metal uptake by maize seedlings (*Zea mays* L.) grown in soils spiked with Lanthanum and Cadmium. Environ. Pollut..

[26] Netty S., Wardiyati T., Maghfoer M.D., Handayanto E. (2013). Bioaccumulation of nickel by five wild plant species on nickel-contaminated soil. J. Eng..

[27] Neina D. (2019). The role of soil pH in plant nutrion and soil remediation. Appl. Enviroental Soil Sci..

[28] Clemente R., Piechur D.J., Bernal M.P. (2003). Uptake of heavy metals and As by *Brassica juncea* grown in a contaminated soil in Aznalcóllar (Spain): The effect of soil amendments. Environ. Pollut..

[29] Xu J.M., Tang C., Chen Z.L. (2006). The role of plant residues in pH change of acid soils differing in initial pH. Soil Biol. Biochem..

[30] Gajic G., Djurdjević L., Kostić O., Jarić S., Mitrović M., Pavlović P. (2018). Ecological potential of plants for phytoremediation and ecorestoration of fly ash desposits and mine wastes. Front. Environ. Sci..

[31] Tsonev T., Lidon F.J.C. (2024). Zinc in plants—A overview. Emir. J. Food Agric..

[32] Broadley M.R., White P.J., Hammond J.P., Zelko I., Lux A. (2007). Zinc in plants. New Phytol..

[33] Mir A.R., Pitchel J., Hayat S. (2021). Copper:uptake, toxicity and tolerance in plants and managament of Cu-contaminated soil. BioMetals.

[34] Kumar V., Pandita S., Sidhu G.P.S., Sharma A., Khanna K., Kaur P., Bali A.S., Setia R. (2021). Copper bioavailability, uptake, toxicity and tolerance in plants: A comprehensive review. Chemosphere.

[35] Asare M.O., Száková M.O., Tlustoš P. (2022). The fate of secondary metabolites in plants growing on Cd-, As-, and Pb-contaminated soils- a comprehensive review. Environ. Sci. Pollut. Res..

[36] Haider F.U., Liqun C., Coulter J.A., Cheema S.A., Wu J., Zhang R., Wenjun M., Farooq M. (2021). Cadmium toxicity in plants: Impacts and remediation strategies. Ecotoxicol. Environ. Saf..

[37] Bali A.S., Sidhu G.P.S. (2021). Arsenic acquisition, toxicity and tolerance in plants- from physiology to remediation: A review. Chemosphee.

[38] Zulfigar U., Farooq M., Hussain S., Maqsood M., Hussain M., Ishfaq M., Ahmad M., Anjum Z. (2019). Lead toxicity in plants: Impacts and remediation. J. Environ. Manag..

[39] Mleczek P., Borowiak K., Budka A., Niedzielski P. (2018). Relationship between concentration of rare earth elements in soil and their distribution in plants growing near a frequented road. Environ. Sci. Pollut. Res..

[40] He H., Fan C., Peng Q., Wu M., Zheng J., Wu G.L. (2019). Bioaccumulation and translocation of rare earth elements in two forage legumes grown in soils treated with coal fly ash. Chem. Geol..

[41] Le Jean M., Montargès-Pelletier E., Rivard C., Grosjean N., Chalot M., Vantelon D., Spiers K.M., Blaudez D. (2023). Locked up inside the vessels: Rare rarth elements are transferred and stored in the conductive tissues of the accumulating fern *Dryopteris Erythrosora*. Environ. Sci. Technol..

[42] Liang T., Li K., Wang T. (2014). State of rare earth elements in different environmental components in mining areas of China. Environ. Monit. Assess..

[43] Yuan M., Liu C., Liu W., Guo M., Morel J.L., Huot H., Yu H., Tang Y., Qiu R. (2018). Accumulation and fractionation of rare earth elements (REEs) in the naturally grown Phytolacca americana L. in southern China. Int. J. Phytoremediation.

[44] Liu W., Zheng H., Liu C., Guo M., Zhu S., Cao Y., Qiu R., Morel J.L., van der Ent A., Tang Y. (2021). Variation in rare earth element (REE), aluminium (Al.) and silicon (Si) accumulation among populations of the hyperaccumulator Dicranopteris linearis in southern China. Plant Soil.

[45] Challaraj E.S., Anandkumar B., Natesan M., Maruthamuthu S. (2019). Efficacy of rare earth elements on the physioloical and biochemical characteristics of *Zea mays* L.. Aust. J. Crop Sci..

[46] Carpenter D., Boutin C., Allison J.E., Parsons J.L., Ellis D.M. (2015). Uptake and effects of six rare earth elements (REEs) on selected native and crop species growing in contaminated soils. PLoS ONE.

[47] Krgović R., Trifković J., Milojković-Opsenica D., Manojlović D., Marković M., Mutić J. (2015). Phytoextraction of metals by *Erigeron canadensis* L. from fly ash landfill of power plant “Kolubara”. Environ. Sci. Pollut. Res..

[48] Zhuang P., Yang Q.W., Wang H.B., Shu W.S. (2007). Phytoextraction of heavy metals by eight plant species in the field. Water Air Soil Pollut..

[49] Tyler G. (2004). Rare earth elements in soil and plant systems-A review. Plant Soil.

[50] Xiaoquan S., Haiou W., Shuzhen Z., Hanfa Z., Yan Z., Hong Y., Bei W. (2003). Accumulation and uptake of light rare earth elements in a hyperaccumulator *Dicropteris dichotoma*. Plant Sci..

[51] Ozaki T., Enomoto S., Minai Y., Ambe A., Ambe F., Makide Y. (2000). Beneficial effect of rare earth elements on the growth of *Dryopteris erythrosora*. J. Plant Physiol..

[52] Cakaj A., Hanć A., Lisiak-Zielińska M., Borowiak K., Drapikowska M. (2023). *Trifolium pratense* and the heavy metal contect in various urban areas. Sustainability.

[53] Zhang C., Geng N., Dai Y., Ahmad Z., Li Y., Han S., Zhang H., Chen J., Yang J. (2023). Accumulation and distribution characteristics of rare earth elements (REEs) in the naturally grown marigold (*Tagetes erecta* L.) from the soil. Environ. Sci. Pollut. Res..

[54] Azizi M., Faz A., Zornoza R., Martinez-Martinez S., Acosta J.A. (2023). Phytoremediation potential of native plant species in mine soils polluted by metal(loid)s and rare earth elements. Plants.

[55] Kotelnikova A.D., Ragova O.B., Stolbova V.V. (2021). Lanthanides in the Soil: Routes of Entry, Content, Effect on Plants, and Genotoxicity (a Review). Degrad. Rehabil. Conserv. Soils.

[56] Antoniadis V., Shaheen S.M., Stark H.J., Wennrich R., Levizou E., Merbach I., Rinklebe J. (2021). Phytoremediation potential od twelve wild plant species for toxic elements in a contaminated soil. Environ. Int..

[57] Sasmaz M., Akgul B., Yildirim D., Sasmaz A. (2016). Mercury uptake and phytotoxicity in terrestrial plants grown naturally in the Gumuskoy (Katahya) mining area, Turkey. Int. J. Phytoremedation.

[58] Khashj S., Karimi B., Makhdoumi P. (2018). Phytoremediation with *Festuca arundinacea*: A mini review. J. Health Rep. Technol..

[59] Albornoza C.B., Larsen K., Landa R., Quirga M.A., Najle R., Marcovecchio J. (2016). Lead and zinc determinations in *Festuca arundinacea* and *Cyndon dactylon* collected from contaminated soils in Tandil (Buenos Aires Provinces), Argentina. Environ. Earth Sci..

[60] Steligia T., Kluk D. (2020). Application of *Festuca arundinacea* in phytoremediatin of soils contaminated with Pb, Ni, Cd and petroleum hydrocarbons. Ecotoxicol. Environ. Saf..

[61] Batty L.C., Anslow M. (2008). Effect of a Polycyclic Aromatic Hydrocarbon on the phytoremediation of zinc by two plants species (*Brassica juncea* and *Festuca Arundinacea*). Int. J. Phytoremediation.

[62] Ziarati P., Vambol V., Vambol S. (2019). Use of inductively coupled plasma optical emission spectrometry detection in determination of arsenic bioaccumulaition in *Trifolium pratense* L. from contaminated soil. Ecol. Quest..

[63] Manke J., Praspaliauskas M., Pedisius N., Sujetoviene G. (2024). Evaluation of phytoremediation efficiency of shooting range soil using the bioaccumulation potential and sensitivity of different plant species. Ecol. Eng..

[64] Zand A.D., Muhling K.H. (2022). Phytoremediation cability and copper uptake of maize (*Zea mays* L.) in copper contaminated soils. Pollutans.

[65] Rizwan M., Ali S., Qayyum M.F., Ok Y.S., Zia-ur-Rehman M., Abbas Z., Hannan F. (2017). Use of maize (*Zea mays* L.) for phytomanagement of Cd-contaminated soils: A critical review. Environ. Geochem. Health.

[66] Boros-Lajszer E., Adiloglu S., Goker M. (2021). Phytoremediation: Elimination of hexavalent chromium heavy metal using corn (*Zea mays* L.). Cereal Res. Commun..

[67] Wyszkowska J., Kucharski J. (2021). Phytoremediation of soil contaminated with nickel, cadmium an cobalt. Int. J. Phytoremediation.

[68] Jean M.L., Grosjean N., Spiers K., Rivard C., Montarges-Pelletier E., Flayac J., Gross E., Thomine S., Merlot S., Blaudeza D. Ferns for rare earth elements (REE)- toward deciphering REE transfer to plants using the accumulating fern *Dryopteris erythrosora*. Proceedings of the Final Conference of the COST NOTICE TD1407: Network on Technology-Critical Elements- from Environmental Proces to Human Healt Threats.

[69] Madanan M.T., Shan I.K., Varghese G.K., Kaushal R.K. (2021). Application of Aztec marigold (*Tagetes erecta* L.) for phytoremediation of heavy metal polluted lateritic soil. Environ. Chem. Ecotoxicol..

[70] (1998). Soil quality—Determination of total nitrogen content by dry combustion (“elemental analysis”).

[71] (1995). Soil quality—Determination of organic and total carbon after dry combustion (elementary analysis).

